# Use of Electrical Impedance Tomography to Monitor Regional Cerebral Edema during Clinical Dehydration Treatment

**DOI:** 10.1371/journal.pone.0113202

**Published:** 2014-12-04

**Authors:** Feng Fu, Bing Li, Meng Dai, Shi-Jie Hu, Xia Li, Can-Hua Xu, Bing Wang, Bin Yang, Meng-Xing Tang, Xiu-Zhen Dong, Zhou Fei, Xue-Tao Shi

**Affiliations:** 1 Department of Biomedical Engineering, Fourth Military Medical University, Xi'an, China; 2 Neurosurgical Unit of Xijing Hospital, Fourth Military Medical University, Xi'an, China; 3 Department of Bioengineering, Imperial College London, London, United Kingdom; Hungarian Academy of Sciences, Hungary

## Abstract

**Objective:**

Variations of conductive fluid content in brain tissue (e.g. cerebral edema) change tissue impedance and can potentially be measured by Electrical Impedance Tomography (EIT), an emerging medical imaging technique. The objective of this work is to establish the feasibility of using EIT as an imaging tool for monitoring brain fluid content.

**Design:**

a prospective study.

**Setting:**

In this study EIT was used, for the first time, to monitor variations in cerebral fluid content in a clinical model with patients undergoing clinical dehydration treatment. The EIT system was developed in house and its imaging sensitivity and spatial resolution were evaluated on a saline-filled tank.

**Patients:**

23 patients with brain edema.

**Interventions:**

The patients were continuously imaged by EIT for two hours after initiation of dehydration treatment using 0.5 g/kg intravenous infusion of mannitol for 20 minutes.

**Measurement and Main Results:**

Overall impedance across the brain increased significantly before and after mannitol dehydration treatment (*p* = 0.0027). Of the all 23 patients, 14 showed high-level impedance increase and maintained this around 4 hours after the dehydration treatment whereas the other 9 also showed great impedance gain during the treatment but it gradually decreased after the treatment. Further analysis of the regions of interest in the EIT images revealed that diseased regions, identified on corresponding CT images, showed significantly less impedance changes than normal regions during the monitoring period, indicating variations in different patients' responses to such treatment.

**Conclusions:**

EIT shows potential promise as an imaging tool for real-time and non-invasive monitoring of brain edema patients.

## Introduction

Cerebral edema is a clinical condition with excess accumulation of fluid in the intracellular or extracellular space of the brain and a common emergency condition in neurology. It has become increasingly evident that the formation of cerebral edema is one of the major factors leading to the high mortality and morbidity in affected individuals. Indeed, some studies have reported that cerebral edema may account for up to half of the mortality in all victims of traumatic brain injury [1]. Edema causes cell swelling that alters cellular metabolite concentration and consequently cellular physiology, biochemistry, and function. Such swelling can also cause a rapid increase in intracranial pressure (ICP), which results in compression of blood vessels, reduction of tissue blood flow, reduction of oxygenation, and eventually shift of tissue down pressure gradients (herniations) that may damage vital brain parts involved in respiration and cardiac function [2].

Currently there is a need for a dynamic technique to monitor the development and treatment of brain edema in real time leading to prompt interventions. At present, CT and MRI are the routine diagnostic methods for cerebral edema [3]. However, these imaging techniques cannot be used for continuous monitoring and cannot reflect real-time effects of any treatment [4]. Although ICP monitor is able to record the ICP variation in real time and to indicate the development of cerebral edema, the technique requires surgical procedures to invasively establish a sensor into the meninges and the cost of such disposable sensor is high [5]. A safe, low-cost, real-time, and non-invasive imaging tool sensitive to the amount of fluid within brain for monitoring the development and treatment of cerebral edema is highly desirable in clinical practice.

Electrical impedance tomography (EIT) is an emerging medical imaging technique that seeks to determine internal electrical impedance distributions within the body by injection of electrical currents and measurement at electrodes on the surface of an area of interest [6]. Safe electrical currents (e.g. typically 1 mA at 50 kHz) are injected into the object and the corresponding boundary potentials are measured by a predefined set of electrodes. A cross-sectional EIT image is then reconstructed from these measurements. If two sets of measurements are generated at two different time points, then a difference image of the relative changes in resistivity can be reconstructed. This is referred to as “difference EIT”, which is less affected by some common sources of errors as they are cancelled out between the two measurements.

Being noninvasive and safe from ionizing radiation, EIT has been proposed for medical applications such as breast cancer detection [7], assessing gastrointestinal conditions or abdominal bleeding [8] and imaging of the ventilation and perfusion distribution in the thorax [9]. EIT for ventilation performance monitoring system has been approved and clinically available in Europe [10].

Application of EIT to brain is more challenging due to the high impedance posed by skull and the shunting of current by the scalp [11]. It is also the case that the considerable variations between patients in skull thickness and variability of skull thickness in one patient make accurate EIT imaging very difficult. Some initial studies were conducted either with exposed brain or on neonatal brain where skull impedance is less. A group at University College London (UCL) first demonstrated that the UCLH Mark EIT system could generate reproducible EIT images of epileptic seizures, functional activity, and the phenomenon of spreading depression in anaesthetized experimental animals with a ring of electrodes on exposed brain [12], [13]. Later on the same group demonstrated the feasibility of imaging adult brain function non-invasively on healthy volunteers and epileptic patients [14], [15]. Meanwhile, a group at University of Florida had detected intraventricular hemorrhage (IVH) in neonatal piglets using EIT [16]. Our group has a research focus on developing EIT as an image monitoring tool for brain diseases. We have previously demonstrated that subarachnoid hemorrhage and intracerebral hemorrhage in neonatal piglets could be detected by EIT and hemorrhage blood volume correlated with magnitude of impedance changes [17], [18]. We also demonstrated clinically that electrical potential changes measured on the scalp are related to changes in local brain impedance introduced by surgical procedures [19].

As the physiological fluid contains highly conductive ions, changes in fluid content of the tissue in brain would cause changes in tissue impedance which makes EIT a potential image monitoring tool for brain edema. In this study EIT is used, for the first time as far as we are aware, for real-time and non-invasive imaging and monitoring of brain impedance changes due to variation of cerebral fluid content during clinical dehydration.

## Methods and Materials

### Ethics Statement

The study was approved by the Fourth Military Medicine University Ethics Committee on Human Research and informed written consent was obtained from those patients' nearest relatives.

### 1. Patients

In this study, 30 patients with cerebral edema were recruited. Of these patients, the data from 7 cases were unavailable in the study due to excessive patient motion and/or serious clinical symptoms, and the remaining 23 cases successfully completed the experiments. These 23 patients' information is summarized as follows: male (11), female (12), age: mean = 54.7 (range 36–85), Glasgow Coma Scale: mean = 7.36 (range 3–15), lesion type: stroke 16, trauma 7; lesion location: lobar 11, Basal/thalamic ganglia 6, other location 6.

Mannitol dehydration treatment has been shown to be effective in ameliorating brain edema and intracranial hypertension and is routinely used in brain edema patients in the Neurosurgical Unit of Xijing Hospital, Fourth Military Medical University, Xi'an, China. Since it is a clinical procedure that is readily available and its effect on brain water content is controllable and predictable [19], it is chosen as a clinical model to study the feasibility of EIT in monitoring brain water content in brain edema.

Patients with one of the following conditions were excluded from the study: patients with severe scalp and skull damage, easily agitated patients, patients with cardiac pacemaker or other metal implants, and patients in critical conditions.

### 2. Electrical impedance tomography and the system evaluation

EIT data were measured in real time using an EIT monitoring system (FMMU-EIT5) developed by our group for brain imaging [20]. The system consists of 16 electrodes. Electrical currents were driven in turn through pairs of electrodes opposite each other and voltages on other adjacent electrode pairs were measured. The working frequency of the system ranges from 1 kHz to 190 kHz, the current from 500 uA to 1250 uA with a measuring accuracy at ±0.01% and the common mode rejection ratio over 80 dB. In this study 1 mA–50 kHz alternating current was used. Repeated measurements were made and averaged before image reconstructed. The reconstruction algorithm is the damped least square method [21], taking into account the realistic boundary shape of the phantom in a finite element model. A single CT scan made before the dehydration treatment for each patient was used as an anatomical reference, on which the EIT images were overlaid.

The EIT system was firstly evaluated by imaging different agar objects of known resistivity within a human head mimicking phantom consisting of an artificial skull of appropriate shape and resistivity in a saline-filled tank [19]. The skull phantom was made of dental plaster with physiologically relevant resistivity distribution [22].To evaluate the spatial resolution, an agar cylinder of 0.3 cm diameters with 50% greater in impedance than saline was measured at six different positions. The phantom without the agar object was measured as a reference for difference imaging. The Full Width at Half Maximum (FWHM) of the object in the image was calculated to quantify the spatial resolution. To evaluate the system impedance sensitivity, agar cylinders of 2 cm diameters with various impedance (from 80% below to 80% above the saline background impedance) was measured at a fixed position of the phantom.

### 3. Clinical experimental protocol

It should be noted that in this study no changes in clinical treatment and management protocol for the selected patients were made. The only addition to the existing clinical protocol in this study was the measurements of EIT data.

#### 3.1 Positioning of electrodes

16 EIT electrodes were evenly fixed on an elastic belt and placed around the patient's shaved head to form a circular plane. Each electrode was coupled to scalp via conductive gel to reduce the contact impedance.

#### 3.2 Injection of mannitol and EIT monitoring

For each patient 0.5 g/kg of mannitol solution was administered via intravenous infusion in 20 minutes. EIT data acquisition was started 30 minutes before mannitol injection and lasted for at least 150 minutes, with a frame rate of 1 frame per second. We actually acquired one data frame by averaging the current 10 frames in order to reduce the measurement noise by 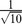. Difference EIT images were reconstructed with a reference data set before mannitol administration. In image reconstruction each individual patient's head shape, obtained from segmenting the corresponding CT image, was taken into account in a finite element model.

### 4. EIT data analysis

**Figure 1:**
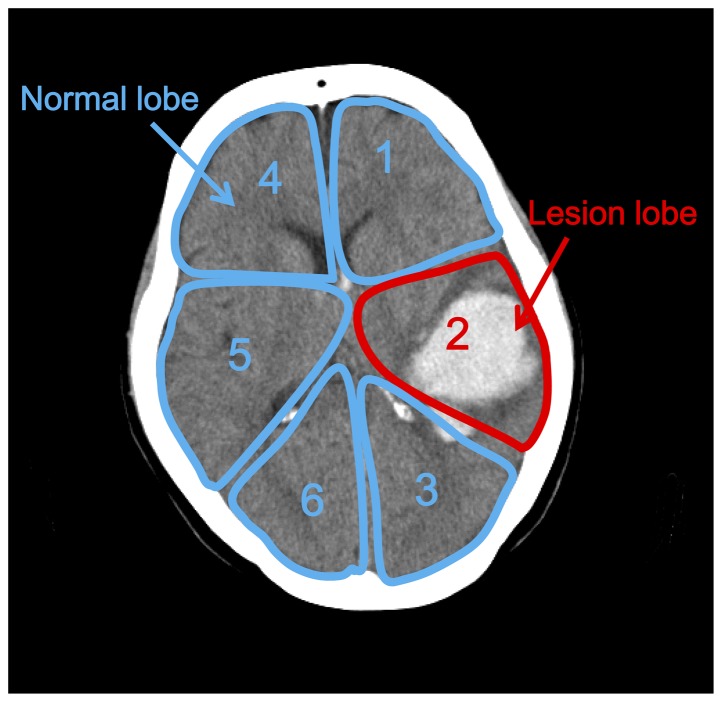
The definition of Regions of Inerest (ROIs). The brain is segmented into six lobes according to anatomy, including left frontal lobe, right frontal lobe, left temporal lobe, right temporal lobe, left occipital lobe and right occipital lobe. The six lobes are then classified into normal lobes and lesion lobe according to corresponding CT images for subsequent impedance observation.

Firstly each patient's brain images were divided into six regions of interest (ROI): left frontal lobe, right frontal lobe, left temporal lobe, right temporal lobe, left occipital lobe, and right occipital lobe (Fig. 1). Next, each of the six regions was classified as either a normal or a lesion lobe. Finally, the average impedance changes both for the whole brain and for these two individual classes, normalized by the maximum impedance value during the course of treatment, were calculated. In statistical analysis, paired-*t* test was used to evaluate any significant difference in impedance changes before and after the mannitol injection and repeated measurement data of ANOVA was used to test the impedance change difference between normal and lesion lobes during the experiments. The significance level was set to *P*<0.01.

### 5. The relationship between ICP and impedance

ICP has been measured on five patients as part of the clinical procedures for monitoring disease progression. EIT data of these five patients were recorded simultaneously in order to initially evaluate the relationship between these two indicators. Before the measurements, the probe of an ICP monitor (HD-58, Codman, UK) was embedded outside the dura of the patients through surgical procedures. During dehydration, both the ICP data recording and EIT measurements were made at the same time. Also, we employed the linear regression analysis to correlate the data of ICP with that of EIT and the paired-t test to assess the changes before and after the dehydration.

## Results

### 1. The EIT system evaluation

**Figure 2:**
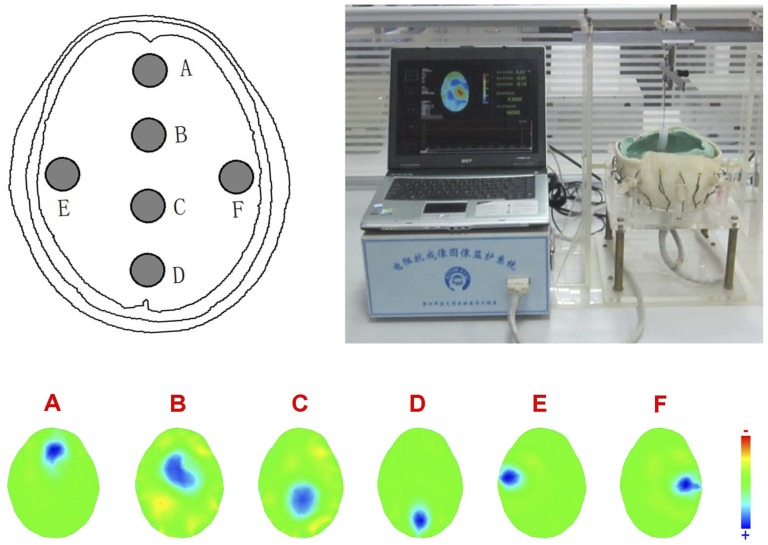
The procedure of EIT validation experiment in realistic head phantom. Top Left: the six pre-defined positions in the phantom. Top Right: a photo of the system calibration experiment. Bottom: The EIT images of an agar cylinder at the six pre-defined positions in the phantom. Note that each image is displayed with its own colorbar in order to show the details.

**Figure 3:**
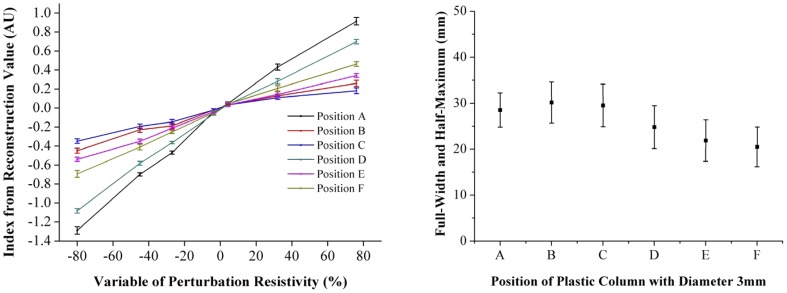
The result of EIT validation experiment in realistic head phantom. The right shows spatial resolutions as a function of spatial location within the phantom. Position E and F (the two positions at 3 and 9 o'clock close to boundary) shows the best spatial resolution among the six positions. The left shows images intensity of the agar cylinders versus their known impedance at the six pre-defined positions. It can be seen that the intensity of EIT images for the agar cylinder is linearly related with its known impedance. The different slope for each spatial position indicates spatially-dependent system sensitivity.

The simulation results demonstrated that the EIT system was able to detect and localize impedance changes due to the introduction of the objects at various positions in the tank (Fig. 2). The FWHM showed that the system's spatial resolution is location-dependent (Fig. 3), a typical feature of an EIT system. Furthermore, the system could also detect the agar cylinders of different impedance and the reconstructed impedance value correlates linearly with the known value (R^2^ = 0.97±0.24) (Fig. 3).

### 2. Clinical results and analysis

**Figure 4:**
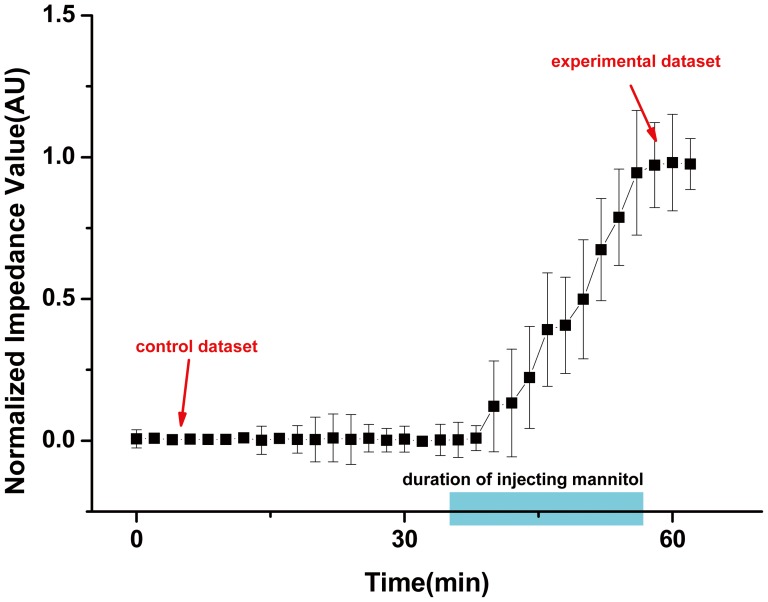
The normalized mean impedance before and after the mannitol injection over time. When the normalized impedance change 30 min before the mannitol injection was chosen as a control dataset, the change 5 min after the injection as an experimental dataset, the impedance of brain significantly increases. The error bar represents SD.

In order to establish whether impedance has changed significantly after mannitol injection for all 23 patients, we used the measurements 30 min before the mannitol injection as control and compared it with those 5 minutes after the mannitol injection. The reconstructed EIT images showed that the overall impedance changes inside the brain increased significantly (*P* = 0.0027) 5 minutes after mannitol injection (Fig. 4).

**Figure 5:**
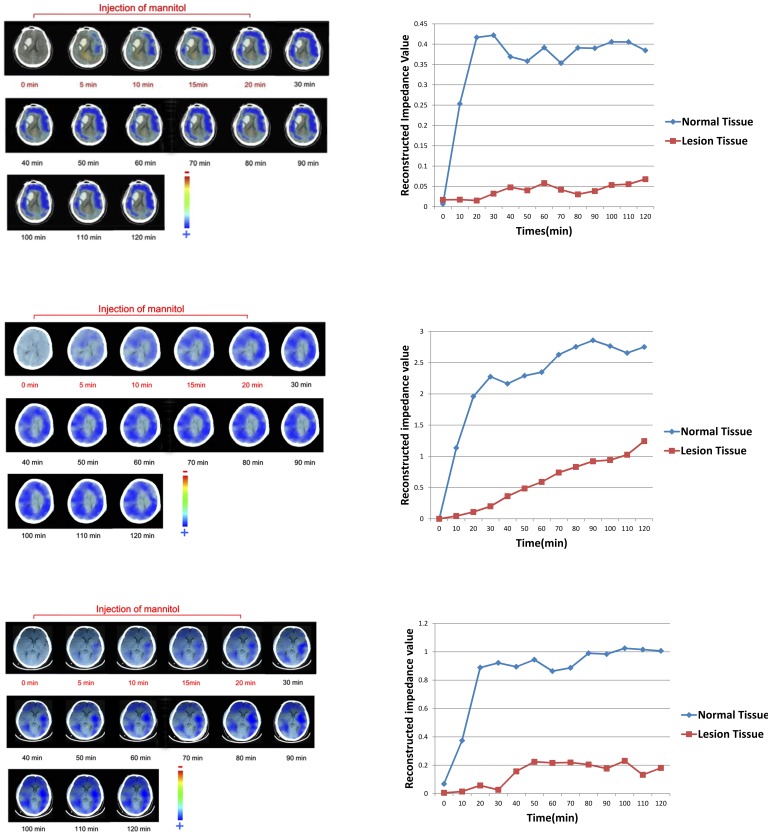
3 out of the 14 patients where, after an initial rise in impedance the impedance remained high.

**Figure 6:**
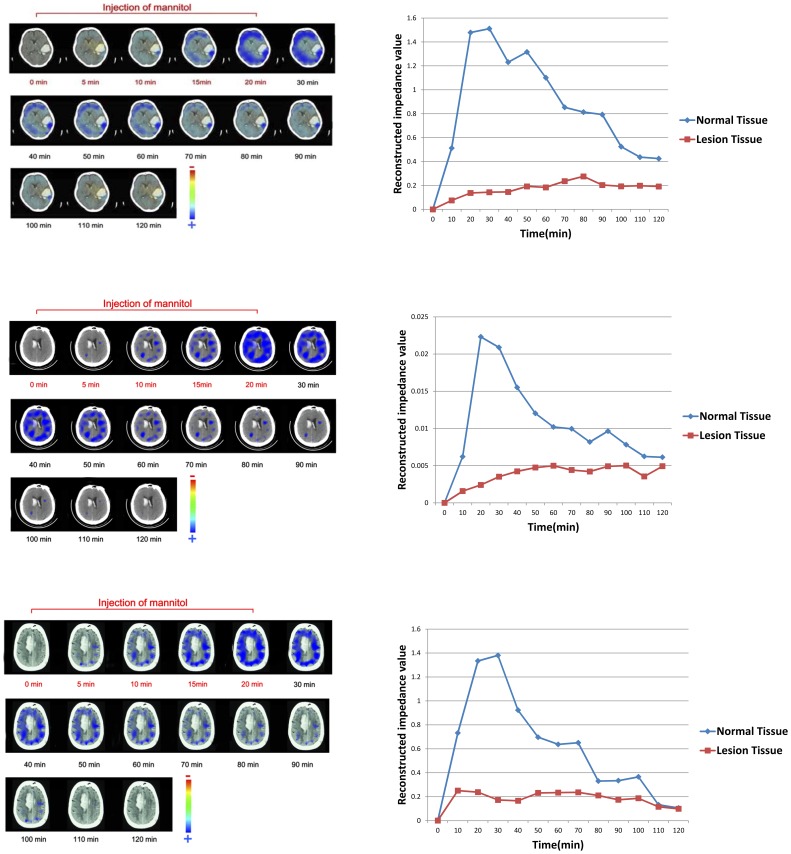
3 of the 9 patients where, after an initial rise in impedance there was a subsequent decrease in impedance.

For further analysis on the EIT image sequences after mannitol injection, there appeared two types of regional impedance changes. Fig. 5 showed 3 cases of the first type (totally 14), in which during the injection the large areas of EIT images became increasingly blue indicating impedance increase in brain, whereas the lesion areas showed much less increase; when the impedance reached the peak after the injection, it remained plateaued for the remainder of the monitoring period. Meanwhile, Fig. 6 showed 3 cases of the other type (totally 9) where the impedance changes start to decrease at the end of injection; two hours after the beginning of the injection, the impedance returned close to its initial value.

**Figure 7:**
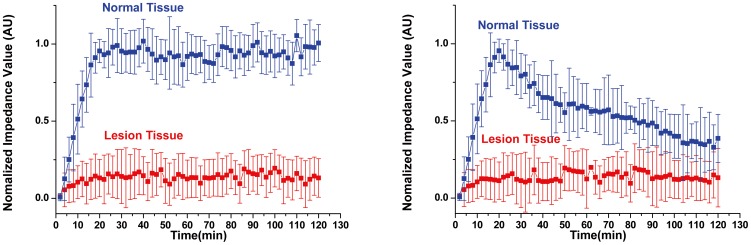
Comparison of impedance variation over time in ROIs with normal tissue versus those with lesion tissue in all 23 patients. **Left:** the first patient group shows that normal tissue promptly increases with the mannitol injection and remains high impedance value after injection (14 cases). **Right:** the second patient group shows the similar result during the injection but the impedance of normal tissue decreases after injection (9 cases). Lesion tissue in both patient groups appears insensitive to dehydration treatment. The error bar represents SD.

For all 23 patients,, the ROI results, along with the analysis of the repeated measurement date of ANOVA, showed that the normalized impedance changes in normal lobes were significantly different from that in lesion lobes (*P* = 0.0032 in the first type, *P* = 0.0089 in the second type). The normalized impedance of normal lobes varied greatly after the injection of mannitol while the normalized impedance of lesion lobes showed much less changes during the same period of time (Fig. 7).

**Figure 8:**
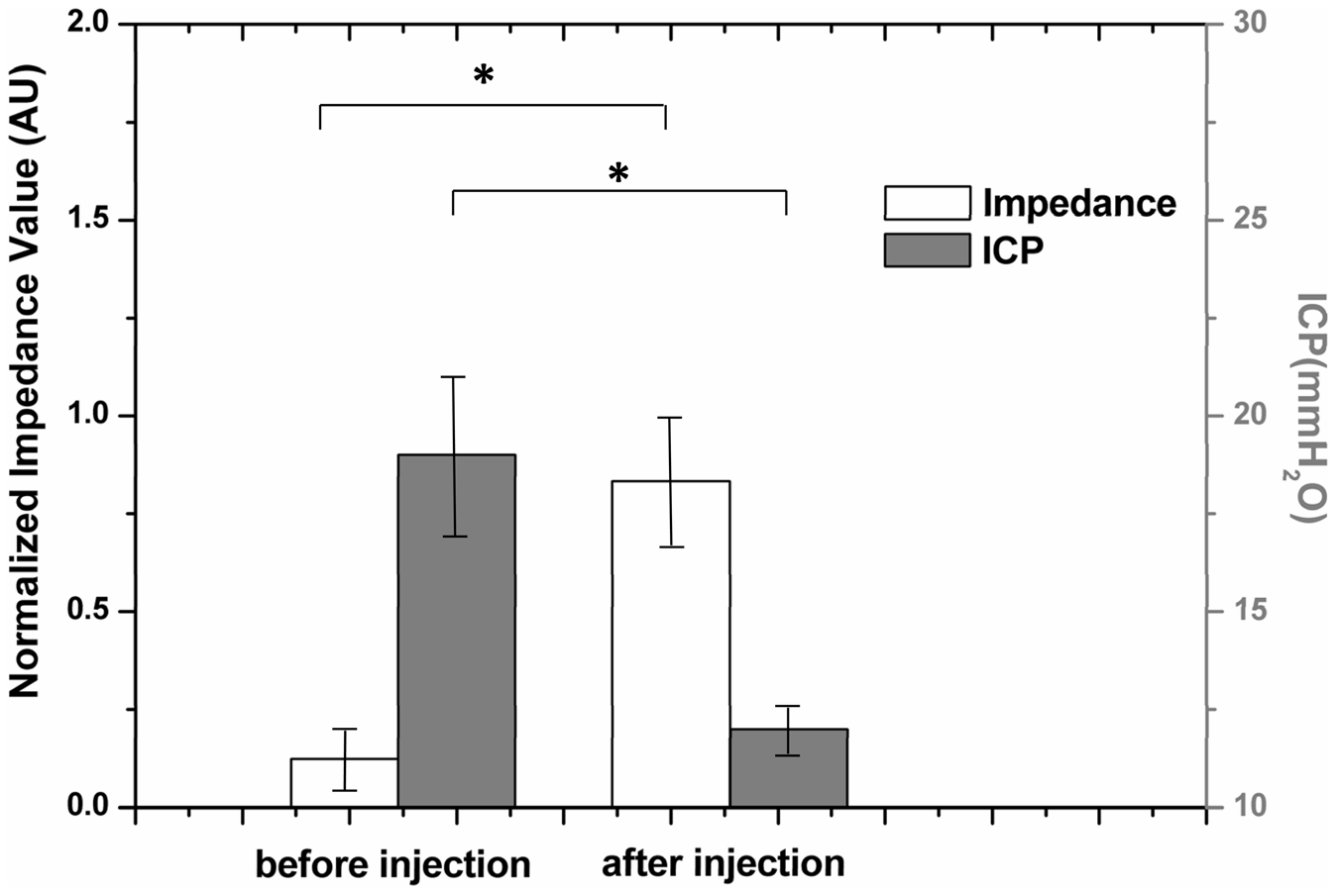
Comparison of EIT measurements and ICP on 5 patients before and after mannital injection. The white bar represents the change of relative impedance, and the grey bar represents the change of ICP, before and after mannitol injection. The difference significance is set to *P*<0.01. The error bar represents SD.

For the five patients who underwent the ICP monitoring, we found that, although the data of ICP and EIT did not show a clear linear correlation, on average the ICP decreased by 32.4% (from 17.9±5.1 to 12.1±1.7 mmH_2_O) and the normalized impedance increased by 531% (from0.13±0.07 to 0.82±0.15) during the dehydration treatment (Fig. 8).

## Discussion and Conclusion

In this paper,EIT was used for the first time, to monitor in real time changes in brain water content in a clinical model with patients undergoing clinical dehydration treatment. The results showed that impedance changes in the EIT images corresponded well to the expected changes of brain fluid content during dehydration, which demonstrated the potential of EIT as an imaging tool for real-time and non-invasive monitoring of brain edema. The EIT results also showed that the effect of mannitol dehydration treatment was more long-lasting in some patients than in others. This is in agreement with previous clinical studies where mannitol treatment alone is said to have different outcomes and could lead to a more personalized treatment [23]. Furthermore, our initial results suggest that different brain tissues have different dehydration effects—normal brain tissues had more significant dehydration than the diseased tissues. This finding agrees with that of Hartwell *et al.* who has found that in models of ischemic infarction, the reduction in brain water content after mannitol infusion is greater in the normal than in the damaged hemisphere [24]. This finding also agrees with that of Videen *et al.* which also showed that volume shrinkage occurred preferentially in the non-infracted hemisphere after mannitol administration [25].

Currently there is a lack of an effective, safe, non-invasive, real-time and low cost monitoring tool for brain edema, a clinical condition which can develop rapidly and be life threatening. ICP monitor is invasive and at high cost. Other imaging modalities such as CT or MRI cannot be used as real-time monitoring tool due to the lack of accessibility, ionizing radiation (CT) and high cost. EIT is an emerging imaging technique which is safe and has low cost. Our initial results shows that our EIT system is able to detect changes in brain fluid content non-invasively and in real-time, and has great potential to become an effective tool for monitoring brain edema patients and detecting early signs of edema development. This could lead to critical and timely intervention and significantly improve the clinical management of patients with brain edema.

Interestingly, we found in this study a phenomenon that mannitol brought about the significant impedance increase in normal brain tissue. The main reason may lay on the fact that the fluid taken away from the brain due to mannitol is highly conductive as it contains ions. The osmotic effect is based on that mannitol cannot cross the cellular membrane or the intact blood-brain barrier (BBB) but fluid within brain tissue containing smaller molecules (H_2_O, Na^+^, K^+^) can. The concentration gradient of mannitol across the vessel wall cause diffusion of fluid containing small molecules such as H_2_O, Na^+^, K^+^ to diffuse into blood [26]. As hypertonic solution, mannitol will greatly increase plasma osmolality after intravenous injection, resulting in rapid diffusion of brain tissue fluid into blood vessels. Finally, with excretion of fluid through kidney cerebral edema is alleviated. Both intracellular and extracellular fluids contain ions such as Na^+^ and K^+^ which are highly conductive and hence loss of such conductive fluids increases the tissue impedance. This explanation is also consistent with previous findings in head trauma that the cerebral impedance is inversely proportional to the tissue water percentage [27]. As for diseased brain tissues, the damaged blood circulation may weaken the dehydration, so EIT images show significantly less impedance changes on lesion lobes.

Furthermore, among two types of brain edema (cytotoxic and vasogenic), cytotoxic edema is essentially a water compartment shift with no change in tissue water content or volume. In contrast, vasogenic edema increases tissue water content, leading to swelling. Tissue swelling thus requires a vascular contribution if it is to occur [28]. The impedance changes in this study due to mannitol in these two types of brain edema might be different as the impedance measurements are more sensitive to interstitial fluid space [29]. Therefore while a significant change in impedance in vasogenic brain edema after mannitol is expected, the changes in cytotoxic brain edema might be more subtle. It may also be slower due to the extra diffusion from cell to the interstitial space.

Besides studying the relationship between cerebral impedance change and dehydration process, we had also conducted some preliminary work on examining the relationship between changes in cerebral impedance and ICP. Though the results did not show strongly linear correlation between the two indexes, probably due to the shortage of samples, the significant increase in impedance as ICP decreases may provide potential for non-invasive monitoring of ICP using EIT. Further studies on these two measures with more patients are required.

It can be seen from the system evaluation results that the EIT system has limitations in its spatial resolution and impedance sensitivity. Firstly the spatial resolution is much worse than existing imaging modalities such as CT or MRI. However it may be argued that spatial resolution is less important in monitoring applications where sensitivity in detecting a warning sign is more critical. Secondly both spatial resolution and sensitivity are spatially variant – being worse towards the centre of the imaging plane. This is a common issue with EIT as current density decay exponentially towards the centre of the imaged region. Therefore, although no direct dehydration existed in central encephalocoele as there were no blood vessels, the lack of sensitivity in the centre of brain with our system means that we were unable to interpret imaging results in the brain centre. One possible solution might be readjustment of electrode distribution so that current density in the central area would be increased.

As far as the study itself concerned, there are two limitations. First, as mannitol dehydrated the brain tissue, it might dehydrate the scalp as well, namely, it might also change the scalp impedance. Hence, it is necessary in future study that one uses the four-terminal approach to simultaneously measure the impedance changes of the scalp during the dehydration, in order to determine how much impedance changes of the scalp influence the impedance changes of the brain. Second, in this initial clinical study, although we had shown that EIT could detect the impedance changes during the mannitol dehydration, we just compared the diseased lobe(s) with the other lobes; in order to apply EIT to localization of evolving pathology, more detailed ROI analysis is needed in a further prospective study where sufficient number of patients with specific brain conditions can be recruited.

Besides, several new quantitative imaging methods of BBB permeability have emerged recently, including near infrared fluorescence, perfusion CT, two-photon microscopy, dynamic contrast enhanced MRI, etc [30], [31], [32], [33]. As BBB permeability highly associated with edema tissue, these methods may provide us new findings about the inherent relationship between cerebral edema and impedance in future studies.

In conclusion, this study has demonstrated, for the first time, detection and imaging of changes in brain water content using EIT in a clinical dehydration model. This is an important step towards developing EIT as a safe, low cost, non-invasive and real-time image monitoring tool for cerebral edema patients.
